# Moral reasoning among Dutch community pharmacists: testing the applicability of the Australian Professional Ethics in Pharmacy test

**DOI:** 10.1007/s11096-019-00869-5

**Published:** 2019-06-28

**Authors:** M. Kruijtbosch, W. Göttgens-Jansen, A. Floor-Schreudering, E. van Leeuwen, M. L. Bouvy

**Affiliations:** 1 SIR Institute for Pharmacy Practice and Policy, Theda Mansholtstraat 5b, 2331 JE Leiden, The Netherlands; 2 Department of Primary and Community Care, Radboud Institute for Health Sciences (RIHS), Radboud University Medical Center, P.O. Box 9101, 120, ELG, 6500 HB Nijmegen, The Netherlands; 3 Division of Pharmacoepidemiology and Clinical Pharmacology, Department of Pharmaceutical Sciences, Utrecht University, P.O. Box 80082, 3508 TB Utrecht, The Netherlands

**Keywords:** Community pharmacists, Moral reasoning, Netherlands, Pharmacy ethics

## Abstract

*Background* Moral reasoning competency is essential in healthcare practice, especially in situations of moral dilemmas when a professional has to choose a morally justifiable action among several suboptimal action options. The Australian Professional Ethics in Pharmacy test (PEP test) measures moral reasoning among pharmacists. In Australia three levels of moral reasoning (schemas) were measured (1) business orientation (2) rules and regulations, and (3) patient rights (i.e. most advanced schema). *Objective* To test the applicability of the PEP test to pharmacists working in the Netherlands. *Setting* Dutch community pharmacy. *Methods* The PEP test consists of 36 statements (items) accompanying 3 moral dilemma scenarios. It was translated into Dutch and completed by 390 pharmacists. Principle component analysis (PCA) was used to investigate construct validity and Cronbach’s Alpha was used to indicate internal consistency of the Dutch version of the PEP test. The eligible grouped statements and perceived possible moral reasoning schemas were compared to the Australian findings. *Main outcome measure* Moral reasoning schemas. *Results* The PCA analysis resulted in 3 components (i.e. possible moral reasoning schemas) that together accounted 27% variance in the data. The statements that represented the moral reasoning schemas ‘business orientation’ and ‘rules and regulations’ were somewhat similar when comparing these with the statements that represented these schemas in the PEP test study. The most advanced moral reasoning schema identified in Dutch pharmacists contained different statements compared to the statements that represented that schema among Australian pharmacists. This schema was labelled ‘professional ethics’. *Conclusion* The PEP test needs further adaptation to the Dutch pharmacy practice context: especially the statements that should reflect the most advanced moral reasoning schema, need more accurate representations of professional pharmacy ethics that guide pharmacists in the Netherlands. Moral reasoning tests for a specific professional setting or country should be developed and adapted by experts who share the same professional values and practice as the respondents.

**Electronic supplementary material:**

The online version of this article (10.1007/s11096-019-00869-5) contains supplementary material, which is available to authorized users.

## Impacts on Practice

- Moral reasoning tests—that aim to test moral reasoning development in health professionals—should be developed and adapted by experts who share the same professional values and professional practice as the respondents.
- Particularly, the most advanced level of moral reasoning of pharmacists is most likely influenced by various aspects such as the national culture regarding pharmaceutical practice and personal values.

## Introduction

Compared to other healthcare practices, like nursing and medicine, ethics receives relatively little attention in pharmacy practice [1–7]. This is surprising considering pharmacists’ worldwide recognition as experts responsible for pharmaceutical care [2, 8]. Like other health professionals, pharmacists experience moral dilemmas in their patient-focussed roles [1, 4, 5, 7, 9–11]. When confronted with such dilemmas, the right thing to do may not be immediately clear. Moral reasoning is needed to make sense of such dilemmas and to make sound ethical decisions [5, 12–14]. This entails reflection on whose and which values are at stake for each of several possible actions as well as consideration of how potential decisions may influence patients’ well-being [12, 15–18]. Competency in moral reasoning implies that a person has the required knowledge and skills to choose actions that are morally justifiable. Moral reasoning is viewed as one of four psychological processes involved in moral (professional) behaviour; the other three processes are moral sensitivity, moral motivation and moral implementation [19–21]. Although there is no sequential relation between these four processes, all are associated with professional behaviour [22–24]. Empirical studies examining health professionals have shown that moral reasoning can, in itself, contribute to clinical competency and improved quality of care [15]. Thus, there is a need for reliable and valid tests that can measure its development in students and health professionals [19, 25].

Moral reasoning development has been measured in pharmacy among both students and practising pharmacists [3, 5, 26–30], predominantly in the US [1, 30] and mainly with the Defining Issues Test (DIT). The DIT, developed by Rest et al. [15], is the most widely used moral reasoning test [15, 19, 31]. The DIT is based on everyday moral scenarios. It was not designed specifically for professional contexts [31]. Tests that are developed for profession-specific contexts may result in more appropriate measures of moral reasoning development in professionals [22, 24, 32]. Thus, Chaar [33] developed the Professional Ethics in Pharmacy test (PEP test, Appendix 1) for community pharmacy in the Australian context.

### Aim of the study

The aim of this study is to test the applicability of the Australian PEP test to Dutch pharmacists.

### Ethics approval and confidentiality

As this study did not include patients who were subjected to a medical intervention, this study was not subject to formal ethical approval according to current Dutch law. All participants gave written informed consent for the use of the collected data for the purpose of the study. No data was collected that could link questionnaire data to individual participating pharmacists.

## Method

The PEP test [33] was developed in analogy to the Defining Issues Test (DIT).

**The Defining Issues Test (DIT)**

The DIT is based on Kohlberg’s cognitive moral development theory [15, 34]. Its short form uses three scenarios that contain different hypothetical moral dilemmas. Each moral dilemma scenario is accompanied by 12 statements that include—to the dilemma related—sentence fragments that can trigger moral reasoning schemas. Such schemas are a person’s beliefs and cognitions in his or her long-term memory of which he or she is not explicitly aware [35]. The sentence fragments—theorised representations of these moral reasoning schemas—function as stimuli of these schemas in a person’s mind. When there are stimuli that resemble previous stimuli and experiences in that person, these can trigger that person’s tacitly preferred moral reasoning schema. Hence, respondents rate and rank the importance of each of the statements to the extent these match their tacitly preferred schema [23, 35]. Three overall moral reasoning schemas have been postulated [15, 34]: the pre-conventional (personal interest) schema, the conventional (maintaining norms) schema, and the post-conventional (principled thinking) schema (Table 1, first column). A person who reasons from a pre-conventional schema is mainly occupied with his or her own interests. A person who reasons from a conventional schema values social norms, laws and regulations. Finally, a person who reasons from a post-conventional schema bases his or her moral reasoning on universal principles such as justice, equality and societal benefit.

**Table 1:** Moral reasoning development schemas of the DIT and PEP test

Cognitive moral development	DIT [15]	PEP test [33]	PEP test [33]	PEP-NL test
	Moral reasoning schemas	Hypothesised moral reasoning schemas of pharmacists in Australia	Definitive moral reasoning schemas of pharmacists in Australia	Definitive moral reasoning schemas of pharmacists in The Netherlands
	(1)	(2)	(3)	(4)
	**Post-conventional**	**Post-conventional**	**Patients’ rights**	**Professional ethics**
	(Beyond personal interest and norms)	(Principles derived from bioethics conveyed in the Australian Code of Ethics: pharmacist as gatekeeper of medications and provider of primary healthcare to the public)	(Statements are related to patients’ rights, whether legal or otherwise)	(Statements are related to pharmaceutical expertise, professional responsibility, counselling/shared decision making, and being professionally autonomous in using knowledge/judgment to care for patients)
	**Conventional**	**Conventional**	**Rules and regulations**	**Rules and regulations**
	(Maintaining norms)	(Regulatory framework: pharmacist becomes entrenched in practice, adopts professional standards)	(Statements are related to legal obligations of the profession)	(Statements are related to legal obligations of the profession)
	**Pre-conventional**	**Pre-conventional**	**Business orientation**	**Business orientation**
	(Personal interest)	(Personal interest: pharmacist at entry level of the profession)	(Statements are related to client care and business viability)	(Statements are related to client care and business viability)

**The PEP test**

The PEP test [33] is derived from the short-form DIT. Like the DIT, it contains three moral dilemma scenarios. These were developed from the context of Australian community pharmacy practice. The first scenario in the PEP test describes a pharmacist who wants to recommend an expensive over-the-counter (OTC) product of uncertain benefit, against a background of mounting financial pressure for the pharmacy (OTC scenario in Appendix 1). The second scenario (morphine scenario in Appendix 1) depicts a client’s request for morphine for her mother, who does not have a prescription. Due to breakthrough pain, this client’s mother currently uses more opiates than prescribed. The request comes at a moment when a doctor is not present to provide the prescription. In the third scenario (repeat prescription scenario in Appendix 1), a pharmacist is asked to approve an early refill of antidepressants for a patient who is going on a holiday. The scenarios are as well accompanied by 12 statements that have to be rated and ranked (Appendix 1). These statements are theorised [33] to trigger three moral reasoning schemas similar to those theorised in the DIT, now adapted to the context of community pharmacists in Australia (Table 1, second column). The theorised pre-conventional, conventional and post-conventional moral reasoning schemas were statistically confirmed in the PEP study. In that study these schemas were respectively labelled as ‘business orientation’, ‘rules and regulations’ and ‘patients’ rights’ (Table 1, third column).

**Translation of the test**

The PEP test was translated into the Dutch language by one member of the research group (WG) and translated back to English by a professional English scientific writer. The translated PEP test (PEP-NL) was tested for face and content validity by the research team and two additional academic health researchers.

**Data collection and data analysis**

This cross-sectional study used the PEP-NL test with Dutch community pharmacists. These pharmacists were either early career pharmacists who completed the PEP-NL test as an assignment at the start of classes on professionalism and pharmaceutical ethics in their postgraduate education or were supervisors of early career pharmacists who completed the test at the start of a course on pharmaceutical ethics. WG distributed and collected the assignments.

There were several control questions included among the 12 statements in order to correct for respondents providing socially desirable answers [15]. If respondents ranked such a control question more than one time, their tests were excluded from the study.

First, a principal component analysis (PCA) was performed to check the PEP-NL rating scores for construct validity. The PEP-NL rating scores were checked for factorability with the Kaiser–Meyer–Olkin’s measure (KMO). This measure should ideally be over 0.6. Subsequently, correlations between variables were tested with Bartlett’s test of sphericity (index *p* < 0.05). Varimax rotation was used to extract the components to increase interpretability. The components were examined by their percentage of variance explained, their eigenvalues (eligible value > 1) and their component statement loadings (eligible value ≥ 0.35) [36]. If statements loaded highly on more than one component (cut-off less than 0.2 difference between components), these were excluded.

Second, Cronbach’s alpha was used to investigate the internal reliability of the remaining eligible statements of each component and the test as a whole. A Cronbach’s alpha equal to or greater than 0.70 was considered reliable. Both the PCA and Cronbach’s alpha calculations were performed using SPSS version 23.

Third, the eligible statements of each component were compared with the eligible statements of each component of the PCA performed in the PEP study [33] and checked against the moral schemas of the PEP test. In case of differences within the clustered statements per component, three members of the research group (MK, AF and MB) examined these statements and labelled, through consensus, a possible new moral reasoning schema. Final consensus on the moral reasoning schemas was reached after a consulting meeting with an expert panel of five senior pharmacists and MK, WG, AF and MB.

## Results

Three hundred ninety respondents (81% early career pharmacists; 19% pharmacist supervisors) completed the PEP-NL test. Fourteen pharmacists (all early career pharmacists) ranked two or more meaningless statements, and their questionnaires were therefore discarded. The PCA was performed for the data of the remaining 376 respondents. Of these respondents, 63% were women and the median age was 27 years (IQR = 25–35 years).

The PCA analysis confirmed the construct validity of the PEP-NL data. The KMO index was 0.74, and the Bartlett test was statistically significant (*p* < 0.000). The scree plot showed small increments in explained variance beyond 5 components. Therefore, the PCA-varimax rotation was performed with 3, 4 and 5 components. The three components explained 27% of the variance in the data and had eigenvalues larger than 2. When the rotation was set at 4 components, the explained variance increased with 5–32% and when set at 5 components with another 4–36%. However, when set at 4 or 5 components, the statements that correlated, did not provide new moral reasoning schemas on top of the first three moral reasoning schemas. In fact, the component with statements that represented ‘rules and regulations’ did split in two, but the statements belonged together as they were all related to aspects of law or regulations related to the profession. The same applied to the component with statements that represented the ‘business orientation’ moral reasoning schema. Therefore, we set the number of components to 3. Table 2 provides the scenario statements’ correlation loadings for the three PCA components. Table 2 shows these loadings per scenario (moral dilemma scenarios 1, 2 and 3, Appendix 1).

**Table 2:** PEP-NL PCA component correlations per three scenario statements

	PEP-NL PCA components		
	(1)	(2)	(3)
*Dilemma 1—OTC scenario*			
**(O1)** Whether you, the pharmacist, are under great financial pressure	0.078	0.384	− 0.370
**(O2)** Whether other pharmacists would approve of such a recommendation	0.268*	0.158*	− 0.162*
**(O3)** Whether you need to offer the client symptom relief to retain her loyalty to the pharmacy	0.180	0.518	− 0.116
**(O4)** Whether the client is a grandmother and not likely to abuse a medication**	0.309*	0.257*	0.153*
**(O5)** Whether there is no criminal offence in selling OTC products in the pharmacy	0.350*	0.238*	0.183*
**(O6)** Whether the Pharmacy Board recently sent out guidelines about Standards of Practice	0.412*	0.198*	0.345*
**(O7)** Whether providing symptom relief to the client will help her feel less discomfort or pain	0.187*	0.284*	0.217*
**(O8)** Whether it is acceptable to appropriate justice in forms amenable to the professional**	0.414*	0.185*	0.007*
**(O9)** Whether a recent article in a reputable journal queried the benefit of that particular OTC for the patient	0.123	− 0.083	0.453
**(O10)** Whether it is fair to persuade a pensioner to pay for an item of uncertain benefit	0.133*	0.135*	0.217*
**(O11)** Whether you don’t want to disappoint her and lose her respect for you	0.156	0.517	0.069
**(O12)** Whether you counsel and explain the options to her as per professional guidelines	0.185	− 0.017	0.456
*Dilemma 2—Morphine scenario*			
**(M1)** Whether you are willing to risk legal ramifications for illegal provision of an opioid to a sick patient	0.535	− 0.103	− 0.097
**(M2)** Whether viability of the business, by complying with patients’ needs, is important	− 0.048	0.451	0.242
**(M3)** Whether the laws of the land are in place to actually protect the public	0.422*	− 0.043*	0.303*
**(M4)** Whether it is a patient’s right to choose to take medicine even if you suspect self-harm	0.147*	0.290*	0.133*
**(M5)** Whether there are strict professional regulations to abide by regardless of circumstances	0.432	− 0.217	0.068
**(M6)** Whether calling for legal advice is appropriate in this situation	0.731	− 0.011	0.075
**(M7)** Whether the ideology of bioethics and civil liberties apply to resource dissemination in general**	0.550*	0.203*	0.126*
**(M8)** Whether it is a pharmacist’s responsibility if a patient forgets to see the doctor in time	0.244*	0.184*	− 0.065*
**(M9)** Whether pain may be controlled by other measures within legal boundaries	0.341*	0.091*	0.099*
**(M10)** Whether your medical indemnity is up to date and renewed	0.566	0.061	− 0.020
**(M11)** Whether you should respond to the trust which the patient has afforded you	0.081*	0.437*	0.333*
**(M12)** Whether the professional and clinical judgment of the pharmacist in this case is relevant	− 0.248	0.274	0.55
*Dilemma 3—Repeat prescription scenario*			
**(R1)** Whether you (the pharmacist) are very busy and need to close shop in half an hour	0.148*	0.319*	− 0.357*
**(R2)** Whether you consider it important to address clients’ needs otherwise business is lost	0.308*	0.356*	− 0.375*
**(R3)** If the patient has a logical reason for requesting supply there is no point in refusing	− 0.060	0.505	− 0.090
**(R4)** Whether it is a patient’s right to choose how and when to take their medicine	0.163	0.564	− 0.043
**(R5)** If the patient is adequately counselled there is no further responsibility for the pharmacist	0.097	0.533	− 0.209
**(R6)** Whether the client’s neighbour is a friend and can be relied upon to report any problems**	− 0.017*	0.490*	0.009*
**(R7)** Whether a citizen is entitled to his or her medicine by law, if prescribed by a doctor	0.520*	0.347*	0.027*
**(R8)** Whether the prescription is legal and ‘‘Immediate Supply’’ is justified and possible	0.325*	0.370*	0.079*
**(R9)** Whether concerns for safety override need for medication	0.059	− 0.035	0.539
**(R10)** Whether it is a pharmacist’s duty to abide by the requirements of the prescription	0.473	0.215	0.035
**(R11)** Whether it is a pharmacist’s duty to exercise professional judgment in dispensing	− 0.081	0.168	0.649
**(R12)** Whether refusing to dispense, since it is not legally due, is the preferred option	0.218*	− 0.085*	0.348*

Underlined scores are eligible PEP-NL component correlations

* Excluded

**Meaningless statement

As illustrated in Table 3, the internal reliability of the three PCA components of the PEP-NL data showed Cronbach’s alpha values of 0.60 (first component), 0.63 (second component) and 0.54 (third component); for the test as a whole, this value was 0.63.

**Table 3:** PCA component reliability of the PEP-NL test

	Number of eligible items	Cronbach’s alpha
Component 1 ‘Rules and regulations’	5	0.60
Component 2 ‘Business orientation’	7	0.63
Component 3 ‘Professional ethics’	5	0.54
Total PEP-NL PCA components	17	0.63

**Comparing eligible statements and schemas**

The comparison of eligible statements per component resulted in two moral reasoning schemas that were also found in the Australian PEP study—‘rules and regulations’ (conventional schema) and ‘business orientation’ (pre-conventional schema)—and in one new moral reasoning schema, which we labelled as ‘professional ethics’ (perceived as a post-conventional schema). The statements that loaded as the ‘professional ethics’ moral reasoning schema deviated completely from the statements that loaded in the PEP study as the post-conventional schema (patients’ rights schema). Table 4 shows the three components and eligible statements.

**Table 4:** Eligible PEP-NL PCA component correlations

Statements	PEP-NL PCA component correlations		
*Component ‘Rules and regulations’*	*(1)*		
**(M6)** Whether calling for legal advice is appropriate in this situation	0.731		
**(M10)** Whether your medical indemnity is up to date and renewed	0.566		
**(M1)** Whether you are willing to risk legal ramifications for illegal provision of an opioid to a sick patient	0.535		
**(R10)** Whether it is a pharmacist’s duty to abide by the requirements of the prescription	0.473		
**(M5)** Whether there are strict professional regulations to abide by regardless of circumstances	0.432		
*Component ‘Business orientation’*		*(2)*	
**(R4)** Whether it is a patient’s right to choose how and when to take their medicine		0.564	
**(R5)** If the patient is adequately counselled there is no further responsibility for the pharmacist		0.533	
**(O3)** Whether you need to offer the client symptom relief to retain her loyalty to the pharmacy		0.518	
**(O11)** Whether you don’t want to disappoint her and lose her respect for you		0.517	
**(R3)** If the patient has a logical reason for requesting supply there is no point in refusing		0.505	
**(M2)** Whether viability of the business, by complying with patients’ needs, is important		0.451	
**(O1)** Whether you, the pharmacist, are under great financial pressure		0.384	
*Component ‘Professional ethics’*			*(3)*
**(R11)** Whether it is a pharmacist’s duty to exercise professional judgment in dispensing			0.649
**(M12)** Whether the professional and clinical judgment of the pharmacist in this case is relevant			0.550
**(R9)** Whether concerns for safety override need for medication			0.539
**(O12)** Whether you counsel and explain the options to her as per professional guidelines			0.456
**(O9)** Whether a recent article in a reputable journal queried the benefit of that particular OTC for the patient			0.453

O (1–12) = Statements of OTC scenario

M (1–12) = Statements of Morphine scenario

R (1–12) = Statements of Repeat prescription scenario

Rules and regulations

As shown in Table 4, the five statements M1, M5, M6, M10 and R10 were considered to represent a moral schema that reflects keeping up with rules and regulations: (M1) ‘whether you are willing to risk legal ramifications for illegal provision of an opioid to a sick patient’, (M5) ‘whether there are strict professional regulations to abide by regardless of circumstances’, (M6) ‘whether calling for legal advice is appropriate in this situation’, (M10) ‘whether your medical indemnity is up to date and renewed’, and (R10) ‘whether it is a pharmacist’s duty to abide by the requirements of the prescription’. In the Australian PEP study, the rules and regulations moral reasoning schema was also identified through statements M1, M5 and R10 but *not* through statements M6 and M10. Statement M6 was excluded from this component in that study because its correlations were too low; statement M10 correlated in that study with statements that represented the business orientation moral schema.

Business orientation

Seven statements (O1, O3, O11, M2, R3, R4, R5) were considered to represent a moral schema that reflects a business orientation (Table 4): (O1) ‘whether you, the pharmacist, are under great financial pressure’, (O3) ‘whether you need to offer the client symptom relief to retain her loyalty to the pharmacy’, (O11) ‘whether you don’t want to disappoint her and lose her respect for you’, (M2) ‘whether viability of the business, by complying with patients’ needs, is important’, (R3) ‘if the patient has a logical reason for requesting supply there is no point in refusing’, (R4) ‘whether it is a patient’s right to choose how and when to take their medicine’, and (R5) ‘if the patient is adequately counselled there is no further responsibility for the pharmacist’. Although this moral reasoning schema was also identified among Australian pharmacists, in the Australian PEP study, statements R3, R4 and R5 correlated with statements that represented the patients’ rights moral reasoning schema.

Professional ethics

The five statements that loaded on this component (O9, O12, M12, R9, R11) were considered to reflect a moral schema labelled as professional ethics (Table 4). These statements are as follows: (O9) ‘whether a recent article in a reputable journal queried the benefit of that particular OTC’, (O12) ‘whether you counsel and explain the options to her as per professional guidelines’, (M12) ‘whether the professional and clinical judgement of the pharmacist in this case is relevant’, (R9) ‘whether concerns for safety override need for medication’, and (R11) ‘whether it is a pharmacist’s duty to exercise professional judgment in dispensing’. In the Australian version of the PEP test, statements O9, O12 and R11 loaded as the rules and regulations moral reasoning schema. Further, statement R9 loaded in the Australian PEP study on the business orientation moral schema component. Statement M12 loaded < 0.3 in that study.

## Discussion

This study shows that the Dutch version of the Professional Ethics in Pharmacy test (PEP test) resulted in two identical moral reasoning schemas compared to the Australian version, and in one different schema, namely the post-conventional moral reasoning schema. However, the PEP-NL test statements need to be adapted to make the test more sensitive to the Dutch community pharmacy context. Such an adapted test would have to be validated once more before it can be applied. This suggests that a similar adaptation and validation process may be needed when applying the PEP test in other countries.

As in the Australian PEP test, our results fit quite well, with the three moral reasoning schemas of the DIT test. We found the pre-conventional level of moral reasoning ‘business orientation’, the conventional level ‘rules and regulations’, and the post-conventional level ‘professional ethics’ (Table 1, fourth column).

As described in the method section, schemas are tacit beliefs and cognitions in the long-term memory of a person. The schemas originate from the specific context wherein that person has lived, worked and still lives and works. The statements of the PEP test are designed to trigger these underlying tacit beliefs and cognitions related to the context of pharmacy practice. For an interpretation of the post-conventional moral reasoning statements of the PEP-NL test and their underlying schema (‘professional ethics’) the context of pharmacy practice in the Netherlands therefore has to be considered.

Pharmaceutical patient care—as a foundational philosophy—was introduced in the 1990s by Hepler and Strand [8] and embraced by the Dutch Pharmaceutical Association [37]. This patient-centred approach and professional practice aims to ensure the effective and safe use of medicines and includes the responsibility for helping patients to achieve definite health outcomes [8, 38]. This pharmaceutical care culture contributed to the development and design of the Dutch *Charter Professionalism of the Pharmacist: Foundation to act professionally and ethically* [39]. The charter states the profession’s core values, which guide pharmacists working in all sectors in the Netherlands. Commitment to the patient’s well-being, which includes protecting the patient’s rights, is an important value, but so are societal responsibility, being reliable and caring, pharmaceutical expertise and professional autonomy [40]. One core value is not more important than another. Keeping in mind this Dutch pharmacy practice context, all statements in the post-conventional schema in the PEP-NL test were interpreted as ‘professional ethics’. For example, the statement (M12) ‘whether the professional and clinical judgement of the pharmacist in this case is relevant’ (morphine scenario, Appendix 1), fits seamlessly with the professional autonomy in moral decision-making, which is expected from Dutch pharmacists. This statement clearly represents the professional responsibility to achieve effective and safe use of medicines in the dilemma concerned. However, the statements can be further refined and adapted to triggers closer to the context. For example, the statement (O12) **‘**Whether you counsel and explain the options to her as per professional guidelines’ would be improved as the text ‘so that the patient can understand and make an informed decision’ was added as trigger for this schema.

Upon interpreting all statements in the pre-conventional level of moral reasoning ‘business orientation’ and the conventional level ‘rules and regulations’ in our PEP-NL test, it was agreed these also exist among pharmacists in the Netherlands. Although, further research is needed to find out if the related statements can be improved further in their function as triggers for these schemas for pharmacists practicing in the health system in the Netherlands.

Australian and Dutch pharmacists seem to share the pre-conventional and conventional schemas of moral reasoning. The majority of the eligible statements that represented these two schemas in our PEP-NL test were the same as those that represented these schemas in the Australian PEP study. This is in contrast to the post-conventional moral reasoning schema in which none of the eligible statements in the PEP-NL test (‘professional ethics’ schema) were the same as the statements that represented the post-conventional schema in the Australian PEP test (‘patients’ rights’ schema). Apparently, different statements representing the post-conventional moral reasoning schema triggered the pharmacists in both countries, suggesting variation in underlying beliefs and cognitions and pharmacy practice context. This is surprising as in both countries pharmacists have a patient-centred pharmaceutical care practice as their highest goal [40, 41]. However, the variation may come from differences in professional guidance (e.g. education, policy) to achieve this patient-centred pharmaceutical care practice and in corresponding professional language [31].

Our results suggest that Dutch pharmacists, when reasoning with the post-conventional moral reasoning schema, are guided by professional ethics as elaborated upon earlier: pharmacists are professionally autonomous in their pharmaceutical responsibility and are socially expected to use their expertise and judgement to provide the best care for the patient. Whereas Australian pharmacists may be educated and guided (professionally) by a more juridical (rights) perspective and thereto related language. The pharmacists may therefore be more focused on performing their legal duties as being the best care for patients and—simultaneously—on avoiding legal consequences [41].

These possible differences in professional guidance and thereto related language may explain why some statements (e.g. R3, R4 and R5) correlated as the post-conventional schema ‘patients’ rights’ in the Australian PEP test, and did not correlate with the statements that appeared in the post-conventional schema in the PEP-NL test.

Similarly, such differences may also explain why the majority of the statements that correlated as the moral reasoning schema ‘professional ethics’ among Dutch pharmacists triggered the ‘rules and regulations’ schema among pharmacists in the Australian PEP test. For example, the statement (O12) ‘whether you counsel and explain the options to her as per professional guidelines’ may have been interpreted by Dutch pharmacists as a professional behaviour because they have internalised the content of the guidelines as ‘good pharmacy practice’, whereas for Australian pharmacists, practising on the basis of guidelines (or laws and regulations) may mean fulfilling one’s (legal) duty [41].

Besides these possible differences in professional guidance and language the variation in underlying beliefs and cognitions may be caused by cross-cultural differences not directly related to the profession. Such cross-cultural differences include the larger context of national socio-economic and healthcare systems, national laws and regulations, religion, family social structures [42–44], and personal values [1, 41]. Therefore, a professional ethics test for a specific professional setting and country should be developed by experts who share the same professional values, practice and language as the respondents [32].

A strength of this study is the number of respondents which allowed us to test applicability with a PCA, because A PCA generally needs at least 300 respondents [36]. A limitation of the study is that the majority of participating pharmacists were early in their careers and therefore not representative of the Dutch pharmacist population in general. Future research should include a more representative cohort-mix of younger and more experienced pharmacists to compare their moral reasoning schemas. However, we as well performed a PCA without the supervisors, who are more experienced pharmacists. That analysis did not result in different moral reasoning schemas.

Another limitation is that the PEP test had to be translated from the Australian into the Dutch language and both countries have different cultural backgrounds and (professional) guidance and language as explained above. For example some referrals to specific institutions mentioned in the PEP test statements had to be adapted and the function of these institutions may be different between countries.

## Conclusion

We conclude that the PEP test, which was originally developed in Australia, needs to be further adapted to the context and professional language of Dutch pharmacy practice. The statements, especially those associated with the post-conventional level of moral reasoning, need adjustments in order to better reflect a moral reasoning schema that is based on professional ethics that guides pharmacists in the Netherlands.

## Electronic supplementary material

Below is the link to the electronic supplementary material.

Supplementary material 1 (DOCX 45 kb)
