# Supplementary material for: Moral reasoning among Dutch community pharmacists: testing the applicability of the Australian Professional Ethics in Pharmacy test
Source: Int J Clin Pharm. 2019 Jun 28;41(5):1323–31. doi: 10.1007/s11096-019-00869-5 (PMC6800840; doi:10.1007/s11096-019-00869-5)
Supplement: Supplementary file 1 — Supplementary material 1 (DOCX 45 kb) [file 11096_2019_869_MOESM1_ESM.docx]

**Appendix 1: Professional Ethics in Pharmacy Test © [33]**

**Dilemma No. 1 (OTC scenario)**

It was a cold winter’s afternoon and business had been slow at the pharmacy all day. In fact business had been slow ever since the pharmacist took over the pharmacy 3 months ago from the previous owner. It had been difficult to keep finances under control at the time. The bank’s notice for late payment instalments had arrived the day before. It was a relief to be distracted from these worries by an incoming client asking to see the Pharmacist. An elderly lady requested something for her sinuses. She had tried many medications including Paracetamol, Antihistamines and nasal sprays, but nothing seemed to have helped. There were many OTC (over-the-counter) products on the shelf with huge bonuses and great promotions. One particularly expensive item looked suitable. Perhaps it might not provide her with much symptom relief, as there was no evidence to

prove efficacy, but it wouldn’t do much harm either.

**Should the pharmacist sell the OTC product?**

Yes; Can’t Decide; No

**How important would each of the following be in deciding what to do?**

Please rate the importance of each of the following by marking with an x:

Great; Much; Some; Little; No

**1**. Whether you (the pharmacist) are under great financial pressure.

**2**. Whether other pharmacists would approve of such a recommendation.

**3**. Whether you need to offer the client symptom relief to retain her loyalty to the pharmacy.

**4**. Whether the client is a grandmother and not likely to abuse a medication.

**5**. Whether there is no criminal offence in selling OTC products in the pharmacy**.**

**6**. Whether the Pharmacy Board recently sent out guidelines about Standards of Practice.

**7**. Whether providing symptom relief to the client will help her feel less pain**.**

**8**. Whether it is acceptable to appropriate justice in forms amenable to the professional.

**9**. Whether a recent article in a reputable journal queried the benefit of that OTC to her.

**10**. Whether it is fair to persuade a pensioner to pay for an item of uncertain benefit.

**11**. Whether you don’t want to disappoint her and lose her respect for you.

**12.** Whether you counsel and explain the options to her as per professional guidelines.

**From the list of questions above, please rank the statements in order of importance**:

Most Important; Second most important; Third most important; Fourth most important

**Dilemma No.2 (Morphine scenario)**

One late Saturday evening a client, well known to the pharmacist, presented at the pharmacy in much distress. Over the last few months she had been collecting her mother’s regular medications for cancer treatment and pain relief. The pharmacist had no doubt the client’s mother was suffering much pain. The client approached the pharmacist imploring for an extra bottle of Morphine Mixture 10mg/mL, as her mother had used up all her repeats and had just run out. The last dispensing of the mixture had been three days earlier. Lately, her mother had needed a lot more morphine than usual for breakthrough pain. The family doctor had left for the weekend. All neighbouring doctors had also left for the weekend and she couldn’t go to the emergency department of the local hospital for this.

**Should the pharmacist dispense the Morphine Mixture?**

Yes; Can’t Decide; No

**How important would each of the following statements be in deciding what to do?**

Please rate the importance of each of the following by marking with an x:

Great; Much; Some; Little; No

**1**. Whether you (the pharmacist) are willing to risk legal ramifications for illegal provision of

an opioid to a sick patient.

**2**. Whether viability of the business by complying with patients’ needs is important.

**3**. Whether the laws of the land are in place to actually protect the public.

**4.** Whether it is a patient’s right to choose to take medication even if you suspect self-harm

**5**. Whether there are strict professional regulations to abide by regardless of circumstances.

**6**. Whether calling for legal advice is appropriate in this situation.

**7**. Whether ideology of bioethics & civil liberties apply to resource dissemination in general.

**8**. Whether it is a pharmacist’s responsibility if a patient forgets to see the doctor in time.

**9**. Whether pain may be controlled by other measures within legal boundaries.

**10.** Whether your medical indemnity is up to date and renewed.

**11.** Whether you should respond to the trust which the patient has afforded you.

**12**. Whether the professional and clinical judgment of the pharmacist in this case is relevant.

**From the list of questions above, please rank the statements in order of importance**:

Most Important; Second most important; Third most important; Fourth most important

**Dilemma No.3: (Repeat prescription scenario)**

It has been a very busy Monday at the pharmacy. There have been a large number of prescriptions, many with problems, then the demanding clients who couldn’t wait for their turn and even lost medications. It has been difficult to maintain order in the pharmacy. In the midst of all this, one client walked in quietly, and was waiting in a corner to be served. Eventually, an assistant brings forth a repeat prescription for his tricyclic antidepressant. The prescription is not due for dispensing for at least another fortnight. The pharmacist queries this and the client shrugs rather despondently, mumbling something about going on a holiday. The pharmacist vaguely remembers this patient…something about an attempted suicide years ago. The prescribing doctor, a psychiatrist, does not like the pharmacist calling during consultation hours as the distraction upsets his patients.

**Should the pharmacist dispense the repeat?**

Yes; Can’t Decide; No

**How important would each of the following be in deciding what to do?**

Please rate the importance of each of the following by marking with an x:

Great; Much; Some; Little; No

**1**. Whether you (the pharmacist) are very busy and need to close shop in half an hour.

**2**. Whether you consider it important to address clients’ needs otherwise business is lost.

**3**. If the patient has a logical reason for requesting supply there is no point in refusing.

**4**. Whether it is a patient’s right to choose how and when to take their medication.

**5**. If the patient is adequately counselled there is no further responsibility for the pharmacist.

**6**. Whether the client’s neighbour is a friend and can be relied upon to report any problems.

**7**. Whether a citizen is entitled to his or her medicine by law, if prescribed by a doctor.

**8**. Whether the prescription is legal and “Immediate Supply” is justified and possible.

**9**. Whether concerns for safety override need for medication.

**10**. Whether it is a pharmacist’s duty to abide by the requirements of the prescription.

**11**. Whether it is a pharmacist’s duty to exercise professional judgment in dispensing.

**12**. Whether refusing to dispense, since it is not legally due, is the preferred option.

**From the list of questions above, please rank the statements in order of importance:**

Most Important; Second most important; Third most important; Fourth most important
